# Supplementary material for: Integrating AI in Pakistani ESL classrooms: Teachers’ practices, perspectives, and impact on student performance
Source: PLoS One. 2025 Sep 30;20(9):e0333352. doi: 10.1371/journal.pone.0333352 (PMC12483258; doi:10.1371/journal.pone.0333352)
Supplement: S1 File — (DOCX) [file pone.0333352.s001.docx]

**Appendix A**

**Pre-test**

**Vocabulary Pre-Test**

**Program:** BS English (3rd Semester)
**Time:** 45 minutes
**Total Marks:** 50

**Section A: Contextual Usage (20 marks)**

*Directions:* Select the most appropriate word from the box to complete each sentence.

**Word Bank:**

ambiguity, rhetoric, dichotomy, pragmatic, paradigm, hegemony, discourse, vernacular, syntax, semantics

1. The ______ shift in literary theory during the 20th century revolutionized textual analysis.
   (Answer: paradigm)
2. Political ______ often employs emotional appeals rather than logical arguments.
   (Answer: rhetoric)
3. The ______ between oral and written traditions remains a key research focus.
   (Answer: dichotomy)
4. Her ______ approach prioritized practical solutions over ideological purity.
   (Answer: pragmatic)
5. Linguistic ______ examines how meaning is constructed in different contexts.
   (Answer: semantics)
6. The novel's deliberate ______ in the final chapter left readers debating multiple interpretations.
   (Answer: ambiguity)
7. Postcolonial studies often analyze how cultural ______ manifests in literature.
   (Answer: hegemony)
8. The professor encouraged students to analyze the political ______ in the presidential debates.
   (Answer: discourse)
9. Regional ______ in the characters' dialogue authenticates the novel's setting.
   (Answer: vernacular)
10. The poet's unconventional ______ challenged traditional grammatical structures.
    (Answer: syntax)

**Section B: Collocations & Phrases (15 marks)**

**Part 1: Academic Collocations** (7.5 marks)
*Match verbs (Column A) with their natural noun partners (Column B):*

| **Column A** | **Column B** |
| --- | --- |
| 1. Cast | a. Precedent |
| 2. Establish | b. Doubt |
| 3. Garner | c. Support |
| 4. Conduct | d. Research |
| 5. Challenge | e. Assumptions |

**Part 2: Academic Phrase Completion** (7.5 marks)
*Select the more natural verb to complete each phrase:*

1. "The text ______ close reading."
   a) requires
   b) demands
2. "This theory ______ further investigation."
   a) warrants
   b) necessitates
3. "The data ______ careful interpretation."
   a) lends itself to
   b) subjects itself to
4. "The findings ______ previous studies."
   a) corroborate
   b) authenticate
5. "The author ______ this argument in Chapter 4."
   a) elaborates
   b) expounds

**Section C: Definitions & Applications (15 marks)**

**Part 1: Term Definitions** (7.5 marks)
*Select the correct definition for each term:*

1. **Hegemony**:
   a) Temporary alliance
   b) Dominant cultural influence *(Correct)*
   c) Economic system
2. **Paradigm**:
   a) A typical example
   b) A conceptual framework *(Correct)*
   c) A moral principle
3. **Discourse**:
   a) Formal discussion of a subject *(Correct)*
   b) Musical rhythm
   c) Visual representation
4. **Pragmatic**:
   a) Idealistic
   b) Practical *(Correct)*
   c) Controversial
5. **Dichotomy**:
   a) Gradual transition
   b) Division into two parts *(Correct)*
   c) Circular argument

**Part 2: Term Application** (7.5 marks)
*Demonstrate understanding by completing these tasks:*

1. Use **"vernacular"** correctly in a linguistics context.
   *(Example: "The study compared the vernacular speech of urban and rural communities.")*
2. Contrast **"syntax"** and **"semantics"** in one sentence.
   *(Example: "While syntax governs sentence structure, semantics deals with meaning.")*
3. Apply **"rhetoric"** to analyze a political speech.
   *(Example: "The politician's rhetoric employed emotional appeals to persuade voters.")*
4. Use **"ambiguity"** to describe a literary device.
   *(Example: "Shakespeare uses deliberate ambiguity in Hamlet's soliloquies.")*
5. Explain **"hegemony"** in media studies context.
   *(Example: "Western media hegemony often marginalizes local narratives.")*

**Post-test**
**Vocabulary Post-Test**
**Program:** BS English (3rd Semester)
**Time:** 45 minutes
**Total Marks:** 50

**Section A: Contextual Usage (20 marks)**

**Directions:** Select the most appropriate word from the box to complete each sentence.

**Word Bank:**
**epistemology**, **lexicon**, **ideology**, **genre**, **intertextuality**, **register**, **metaphor**, **denotation**, **connotation**, **dialect**

1. The film blends elements of several ______ to challenge audience expectations.
2. Academic writing often adopts a formal ______ distinct from everyday conversation.
3. The political speech was saturated with patriotic ______ to stir national sentiment.
4. In literary analysis, ______ refers to the overlap and referencing between texts.
5. His use of the word carried both its literal ______ and a powerful emotional implication.
6. The professor introduced students to basic concepts in ______, the theory of knowledge.
7. Poets frequently rely on ______ to create vivid imagery in their verses.
8. Her research examines the ______ used by youth in urban environments.
9. The novel critiques dominant ______ shaping cultural narratives.
10. The word "home" has a ______ of safety and belonging beyond its literal meaning.

**Section B: Collocations & Phrases (15 marks)**

**Part 1: Academic Collocations (7.5 marks)**

**Match verbs (Column A) with their natural noun partners (Column B):**

| **Column A** |  | **Column B** |
| --- | --- | --- |
| 1. Draw | a. | Inference |
| 2. Propose | b. | Framework |
| 3. Cite | c. | Evidence |
| 4. Offer | d. | Perspective |
| 5. Provide | e. | Justification |

**Part 2: Academic Phrase Completion (7.5 marks)**

**Select the more natural verb to complete each phrase:**

1. "The scholar ______ a new perspective on colonial literature."
   a) puts forward  b) advances
2. "This issue ______ further exploration."
   a) prompts  b) necessitates
3. "The argument ______ from earlier theoretical positions."
   a) stems  b) derives
4. "The conclusion ______ the initial hypothesis."
   a) substantiates  b) supports
5. "The writer ______ a nuanced critique of globalization."
   a) formulates  b) articulates

**Section C: Definitions & Applications (15 marks)**

**Part 1: Term Definitions (7.5 marks)**

**Select the correct definition for each term:**

**Lexicon:**
a) A type of grammar
b) Vocabulary of a language (correct)
c) Cultural narrative

**Intertextuality:**
a) Historical context
b) Relationship between texts (correct)
c) Structural coherence

**Connotation:**
a) Literal meaning
b) Figurative or emotional association (correct)
c) Linguistic function

**Register:**
a) A speech pattern based on age
b) Variation in language based on context (correct)
c) Official documentation

**Metaphor:**
a) Comparison without using "like" or "as" (correct)
b) Scientific explanation
c) Literal description

**Part 2: Term Application (7.5 marks)**

**Demonstrate understanding by completing the following tasks:**

1. Use **"ideology"** in the context of media studies.
   *Example: "The documentary critiques the ideology behind consumer capitalism."*
2. Contrast **"dialect"** and **"register"** in one sentence.
   *Example: "While dialect varies regionally, register shifts based on context and audience."*
3. Apply **"intertextuality"** in literary criticism.
   *Example: "The novel's intertextuality references Shakespeare’s plays to enrich its themes."*
4. Use **"genre"** in a cinematic analysis.
   *Example: "The director’s blend of horror and comedy redefines the genre."*
5. Explain **"epistemology"** in a philosophical context.
   *Example: "Epistemology explores the limits and sources of human knowledge."*

**Writing Pre-Test**

**Program: BS English (Semester 3)
Time: 60 minutes
Total Marks: 50**

**Task 1: Rhetorical Analysis (20 marks)**

***Directions:* Analyze the rhetorical strategies in the provided excerpt from *"A Room of One’s Own"* by Virginia Woolf (300-350 words). Focus on:**

- **Use of ethos/pathos/logos**
- **Structural devices (e.g., parallelism, analogy)**
- **Tone and audience engagement**

**Excerpt Provided**

"Perhaps she scribbled some pages up in an apple loft on the sly, but was careful to hide them or set fire to them. Soon, however, before she was out of her teens, she was to be betrothed to the son of a neighboring wool-stapler. She cried out that marriage was hateful to her, and for that she was severely beaten by her father. Then he ceased to scold her. He begged her instead not to hurt him, not to shame him in this matter of her marriage. He would give her a chain of beads or a fine petticoat, he said; and there were tears in his eyes. How could she disobey him? How could she break his heart? The force of her own gift alone drove her to it. She made up a small parcel of her belongings, let herself down by a rope one summer's night and took the road to London. She was not seventeen. The birds that sang in the hedge were not more musical than she was. She had the quickest fancy, a gift like her brother's, for the tune of words. Like him, she had a taste for the theatre. She stood at the stage door; she wanted to act, she said. Men laughed in her face. The manager—a fat, loose-lipped man—guffawed. He bellowed something about poodles dancing and women acting—no woman, he said, could possibly be an actress. He hinted—you can imagine what. She could get no training in her craft. Could she even seek her dinner in a tavern or roam the streets at midnight? Yet her genius was for fiction and lusted to feed abundantly upon the lives of men and women and the study of their ways. At last—for she was very young, oddly like Shakespeare the poet in her face, with the same grey eyes and rounded brows—at last Nick Greene the actor-manager took pity on her; she found herself with child by that gentleman and so—who shall measure the heat and violence of the poet's heart when caught and tangled in a woman's body?—killed herself one winter's night and lies buried at some crossroads where the omnibuses now stop outside the Elephant and Castle."

**Assessment Criteria**

| **Band** | **Analysis Depth** | **Textual Evidence** | **Academic Style** |
| --- | --- | --- | --- |
| 5 (16-20) | Sophisticated critique of 3+ strategies | Ample, well-chosen quotes | Flawless APA citations |
| 4 (11-15) | Identifies 2 strategies with explanation | Some relevant quotes | Minor citation errors |
| 3 (6-10) | Basic recognition of 1-2 strategies | Limited/poorly chosen quotes | Inconsistent style |

**Task 2: Argumentative Essay (30 marks)**

***Prompt:*
*"Social media platforms democratize public discourse."* To what extent do you agree?** Support your argument with:

- 2 theoretical concepts (e.g., Habermas’ public sphere, echo chambers)
- 1 literary/textual example
- Counterargument and rebuttal

Word Limit: 500-600 words

Rubric

| Criteria | Excellent (25-30) | Proficient (15-24) | Developing (0-14) |
| --- | --- | --- | --- |
| Thesis & Structure | Clear, nuanced claim; logical flow | Defined claim; some organization | Unclear thesis; disjointed |
| Evidence & Analysis | Theoretical + textual support; deep analysis | Adequate support; surface analysis | Weak/no evidence |
| Critical Thinking | Strong counterargument; synthesis | Attempted counterargument | Lacks critical engagement |
| Language & Mechanics | Academic register; minimal errors | Occasional informality/errors | Frequent error |

**Writing Post-Test**

**Program: BS English (Semester 3)
Time: 60 minutes
Total Marks: 50**

**Task 1: Rhetorical Analysis (20 marks)**

**Directions**Analyze the rhetorical strategies in the excerpt below from *A Room of One’s Own* by Virginia Woolf. Your response should be 300–350 words and focus on:

- Use of ethos / pathos / logos
- Structural devices (e.g., repetition, metaphor, parallelism)
- Tone and audience engagement

**Excerpt Provided**

“Intellectual freedom depends upon material things. Poetry depends upon intellectual freedom. And women have always been poor, not for two hundred years merely, but from the beginning of time. Women have had less intellectual freedom than the sons of Athenian slaves. Women, then, have not had a dog’s chance of writing poetry. That is why I have laid so much stress on money and a room of one’s own. A woman must have money and a room of her own if she is to write fiction. That, you will remember, was the title of the paper I was asked to read to you. But, you may say, we asked you to speak about women and fiction—what, has that got to do with a room of one’s own? I will try to explain.

When I came to write this paper I asked myself, what is meant by ‘women and fiction’? There were three meanings I could extract from that phrase. The first is that it is a woman’s position in fiction—how she is represented in books. The second is fiction written by women. And the third—and perhaps the most interesting—is what women are able to write when they are not hindered by law, poverty, or ridicule. But here, I found myself confronted with a problem. It is very difficult to find accurate records of women’s lives. The history of England is a history of the male line—kings, wars, laws, courts. Very little is known about the lives of ordinary women. They left behind no plays, no epics, no monuments. And yet, they lived and felt just as deeply as men. Their reality is not less because it has not been recorded. That absence from history is itself a sign of suppression.

Therefore, it is not just a room, not just money, that women need—it is recognition, representation, and remembrance.”

**Assessment Criteria**

| Band | Analysis Depth | Textual Evidence | Academic Style |
| --- | --- | --- | --- |
| 5 (16–20) | Sophisticated critique of 3+ strategies | Ample, well-chosen quotes | Flawless APA citations |
| 4 (11–15) | Identifies 2 strategies with explanation | Some relevant quotes | Minor citation errors |
| 3 (6–10) | Basic recognition of 1–2 strategies | Limited/poorly chosen quotes | Inconsistent style |

**Task 2: Argumentative Essay (30 marks)**

**Prompt**"Language shapes perception more than perception shapes language."
To what extent do you agree? Support your argument with:

- 2 theoretical concepts (e.g., Sapir-Whorf Hypothesis, Constructivism)
- 1 literary/textual example
- Counterargument and rebuttal

Word Limit: 500–600 words

**Rubric**

| Criteria | Excellent (25–30) | Proficient (15–24) | Developing (0–14) |
| --- | --- | --- | --- |
| Thesis & Structure | Clear, nuanced claim; logical flow | Defined claim; some organization | Unclear thesis; disjointed |
| Evidence & Analysis | Theoretical + textual support; deep analysis | Adequate support; surface analysis | Weak/no evidence |
| Critical Thinking | Strong counterargument; synthesis | Attempted counterargument | Lacks critical engagement |
| Language & Mechanics | Academic register; minimal errors | Occasional informality/errors | Frequent error |

**Appendix B**

**Structured Observation Protocol**

**Study Title: Investigating the Impact of AI-Assisted Instruction on ESL Learners'** Vocabulary and Writing Skills
Observer Name: _____________________
Date: _____________________
Class Level: BS English (Semester 3)
Lesson Focus: Vocabulary / Writing (circle one)
Group: Experimental / Control (circle one)
Duration Observed: __________ minutes

Section A: Classroom Environment and Student Engagement

| Indicator | Description | Rating (1–5)* | Notes |
| --- | --- | --- | --- |
| A1 | Students appear attentive and focused during instruction |  |  |
| A2 | Majority of students participate in learning activities |  |  |
| A3 | Student interactions are relevant to lesson objectives |  |  |
| A4 | Students use AI tools appropriately and independently (if applicable) |  |  |
| A5 | Students demonstrate engagement during writing/vocabulary tasks |  |  |

**Section B: Teacher Practices**

| Indicator | Description | Rating (1–5)* | Notes |
| --- | --- | --- | --- |
| B1 | Teacher clearly explains vocabulary/writing objectives |  |  |
| B2 | Teacher integrates AI tools into instruction (if applicable) |  |  |
| B3 | Teacher monitors and provides feedback during tasks |  |  |
| B4 | Teacher encourages critical thinking and creativity |  |  |
| B5 | Teacher facilitates equitable student participation |  |  |

**Section C: AI Tool Usage *(Only for Experimental Group)***

| Indicator | Description | Rating (1–5)* | Notes |
| --- | --- | --- | --- |
| C1 | AI tools are aligned with lesson goals |  |  |
| C2 | Teacher scaffolds AI tool usage effectively |  |  |
| C3 | AI feedback is used meaningfully by students |  |  |
| C4 | AI tools support vocabulary enhancement |  |  |
| C5 | AI tools enhance the writing process |  |  |

**Section D: Qualitative Field Notes**

Observer comments on student behavior, AI interaction, classroom dynamics, instructional clarity, or any deviations from the planned lesson.

Rating Scale (1–5)

1 = Not Observed
2 = Rarely Observed
3 = Sometimes Observed
4 = Frequently Observed
5 = Consistently Observed

**Appendix C**

**Focus Group Discussion Guide Questionnaire**

1. How do you incorporate AI tools in your teaching practices?
2. What specific benefits have you observed in student performance using AI tools?
3. What challenges have you faced in integrating AI tools into your teaching methods?
4. What training or resources do you think would improve the use of AI in language education?
5. How do you address ethical concerns, such as data privacy, while using AI tools?
