# Supplementary material for: Integrating AI in Pakistani ESL classrooms: Teachers’ practices, perspectives, and impact on student performance
Source: PLoS One. 2025 Sep 30;20(9):e0333352. doi: 10.1371/journal.pone.0333352 (PMC12483258; doi:10.1371/journal.pone.0333352)
Supplement: Attachment — Submitted filename: English Review paper 2.docx [file pone.0333352.s003.docx]

**PONE-D-25-13511: Integrating AI in Pakistani ESL Classrooms: Teachers' Practices, Perspectives, and Impact on Student Performance**

**Recommendation: minor revision**

**A. Abstract Analysis**

The abstract is good in explaining the problem, methodology, results, and final conclusion. At the end of the conclusion, the research findings and the expected follow-up are stated.

**Suggestion:** can be more explicitly strengthened regarding 1) the urgency of the research, especially in the context of theory or practical problems faced; 2) The implementation of the research stage from qualitative data collection techniques (interviews, observations) followed by quantitative analysis methods; 3) Mention the results of quantitative data to strengthen the results obtained;

**B. Introduction Analysis**

**Advantages & Disadvantages**

1) The background of this study is quite clear in describing the importance of the phenomenon being studied, especially in the context of the exploratory sequential mixed-methods method; 2) The author shows the reasons why this study is needed, but it could be strengthened by providing more empirical data or concrete examples that support the urgency of this study; 3) The article cites several related studies but does not analyze the gap between previous studies and this study; 4) If there is a gap between research and previous studies, it needs to be emphasized more explicitly to show the contribution of this study compared to previous studies; 5) The research question is explicit by stating 3 research objectives; 4) There are references to previous studies, but it needs to be more analytical.

**Suggestions:**

1) Can be strengthened by adding supporting data that shows the urgency of this research problem; 2) Analyze the results of previous research more. If the purpose of this research is to fill the gap in previous research, this section can be strengthened by stating the shortcomings or limitations of previous research more explicitly. 3) If necessary, add supporting references and compare more clearly how this research differs from previous studies or to what extent the implications of this research are practical or theoretical; 4) It is better to explain the weaknesses or limitations of previous research that make this research very necessary.

**C. Methodology Analysis**

This study has procedurally carried out the exploratory sequential design stages well. From the methodology, it can be seen that this study uses the results of qualitative analysis to compile quantitative instruments. The process of developing the instrument from the results of interviews or qualitative findings is converted into indicators in a quantitative questionnaire that is tested by expert validity on its quantitative instruments. It's just that it has not been explicitly explained what sampling techniques are used at each stage with the limitations of the sampling techniques used.

**Suggestion:** Explain the sampling technique used.

**D. Analysis of Results and Discussion**

The results presented reflect two stages in exploratory sequential mixed methods, namely the results of the qualitative and quantitative stages.

**Advantages & Disadvantages**

1) This study has a clear objective, in the form of 3 explicit research questions, but in the discussion of the writing, it is not structured how the results of this study answer each research objective explicitly. 2) The interpretation of the table data has not been explained explicitly in the discussion description; 3) This article connects several research results with previous findings but is not sufficient in analyzing the differences or similarities with previous research or the implications of the research results. 4) There are references to supporting theories, but they are not developed enough to clarify the theoretical implications of this study.

**Suggestions:**

1) The writing structure should be clearer in connecting each research result with the research question. 2) Add data interpretation for each table so that readers can understand the relevance of qualitative and quantitative data from the research results. 3) Discussion can highlight more what is new or unique about this research compared to previous research to show the contribution of this research. 4) Discuss whether the results of this research confirm or challenge existing theories and what the practical and theoretical implications of the results of this research are more explicitly.

**E. Conclusion Analysis**

**Advantages & Disadvantages**

Most of the conclusions have answered the research questions, but: 1) The writing structure is not explicit yet in connecting each conclusion of the research results with the previously formulated research objectives, and 2) The research findings are quite clear; there are recommendations for further research that can be carried out based on these findings.

**Suggestion:**

Be more structured in writing the conclusion so that the answers to each research question are clearly.
