# Supplementary material for: Integrating AI in Pakistani ESL classrooms: Teachers’ practices, perspectives, and impact on student performance
Source: PLoS One. 2025 Sep 30;20(9):e0333352. doi: 10.1371/journal.pone.0333352 (PMC12483258; doi:10.1371/journal.pone.0333352)
Supplement: Attachment — Submitted filename: Author Response to Reviewer for AI Manuscript for PLOS ONE.docx [file pone.0333352.s005.docx]

**Author Response to Reviewer #1**

The first reviewer did not suggest any revisions.

**Author Response to Reviewer #2**

We are sincerely grateful to Reviewer #2 for the thoughtful and constructive feedback. We have carefully addressed each point, and the revised manuscript reflects significant improvements in structure, clarity, and scholarly rigor. The revised text for Reviewer 2 is highlighted in GREEN. Below are our point-by-point responses:

**1. Structure and Organization**

a) Introduction too long and theoretical; lacks clear statement of purpose
Response: Thank you for this valuable suggestion. The introduction has been substantially revised to enhance clarity and conciseness. The theoretical background has been condensed and repositioned into the literature review section. Additionally, a clear statement of purpose has been added to the end of the introduction to clarify the study’s unique contribution.

b) Literature review repetition (constructivism, adaptive learning theory)
Response: We appreciate this observation. Redundant explanations in the literature review have been eliminated, and a summary paragraph has been added to streamline the discussion of constructivist and adaptive learning theories.

c) Justification of participant selection (CGPA ≥ 3.5) and generalizability
Response: This concern has been addressed in the sampling section. We now explain the rationale behind selecting high-achieving students (to reduce academic variability) and acknowledge the limitation in terms of generalizability.

d) Results and discussion repetitive; needs more critical analysis
Response: Thank you. We revised the discussion section to reduce repetition and integrate more critical comparisons with existing studies. We have also explicitly connected our findings to relevant literature.

**2. Arguments and Clarity of Contributions**

a) Implications for education policy in Pakistan underdeveloped
Response: A new paragraph in the implications section now outlines concrete policy recommendations for Pakistani education authorities, focusing on AI literacy, infrastructure development, and teacher training.

b) Lack of concrete solutions to AI limitations (e.g., algorithmic bias)
Response: We have expanded our discussion of ethical concerns and algorithmic bias and added practical solutions such as collaboration with local developers and culturally grounded AI training datasets.

c) Impact of AI on teaching methods needs elaboration
Response: We agree with this suggestion. We now include a detailed discussion of how teachers adapted pedagogical strategies in response to AI tools, such as shifting from lecture-based to facilitative instruction.

**3. Data Presentation and Tables**

a) Statistical tables too dense; require clearer interpretation
Response: Each table (Tables 1 and 2) now includes narrative explanations immediately following the data, with clearer interpretation of effect sizes and gains in both control and experimental groups.

b) Suggestion for visualizations
Response (Rebuttal): We appreciate the reviewer’s recommendation. While we agree that visualizations could enhance reader engagement, the manuscript adheres to a journal word limit and format guidelines that prioritize tables over figures. However, all tables are now thoroughly interpreted in accompanying text, providing sufficient clarity for the reader.

Additional Improvements Made

- The data collection process has been expanded to clearly explain how the data were gathered from multiple sources (observations, FGDs, pre/post-tests).
- Clarification that separate tests for vocabulary and writing were designed and implemented (not a single integrated test).

**Author Response to Reviewer #3**

We thank Reviewer #3 for the detailed and constructive feedback. Below, we address each point raised and outline the specific revisions made to improve the manuscript. The revised text for reviewer 3 is highlighted in RED.

**A. Abstract Analysis**

**Reviewer Suggestion**

Strengthen the abstract in terms of urgency, methodological clarity (qualitative followed by quantitative), and explicit reporting of quantitative findings.

**Author Response**
We appreciate the reviewer’s suggestion. In response, we have revised the abstract to:

- More explicitly state the practical and theoretical urgency of integrating AI in Pakistani ESL classrooms.
- Clarify the exploratory sequential design, noting that qualitative data collection (observations and FGDs) preceded the development and implementation of quantitative pre- and post-tests.
- Include specific quantitative results such as gain percentages and t-test outcomes for both vocabulary and writing scores, enhancing the empirical rigor of the abstract.

**B. Introduction Analysis**

**Reviewer Suggestion**

Provide empirical data to support the urgency of the study; clarify the gap with prior research; analyze previous studies more critically.

**Author Response:**
We have significantly strengthened the introduction by:

- Adding empirical indicators such as Pakistan’s digital readiness post-COVID-19 and the Higher Education Commission’s directives for AI adoption.
- Clarifying the research gap by contrasting existing literature, which often emphasizes AI’s affordances in advanced contexts, with the lack of grounded studies in under-resourced ESL environments.
- Providing more critical engagement with cited works to highlight where this study extends, challenges, or complements previous research.
- Clearly positioning the current study as a response to limitations in prior literature—particularly the lack of integration between contextual challenges and pedagogical application in Pakistani ESL settings.

**C. Methodology Analysis**

**Reviewer Suggestion**

Explain the sampling techniques used at each stage of the study.

**Author Response:**
We thank the reviewer for pointing this out. We have now:

- Explicitly stated that purposive sampling was used in the qualitative phase to select experienced AI-trained ESL teachers and that convenience sampling with a CGPA criterion was employed for selecting students in the quantitative phase.
- Clarified that these students were then randomly assigned to experimental and control groups to ensure internal validity.
- Acknowledged the limitations of these sampling techniques in terms of generalizability, and explained how we mitigated bias by balancing demographic variables across groups.

**D. Results and Discussion Analysis**

**Reviewer Suggestion**

Ensure results are explicitly linked to research questions; interpret quantitative data clearly; emphasize theoretical contributions and unique findings.

**Author Response:**
We have revised the Results and Discussion sections to:

- Use headings/subsections that correspond directly to each of the three research questions, thus improving structural clarity.
- Provide clear interpretations of all tables and statistics, including percentage gains, significance levels, and effect sizes.
- Enhance the discussion of novelty, particularly:
  - The cultural-linguistic misalignment between AI feedback and Pakistani English norms;
  - Teacher roles as mediators of AI efficacy;
  - The blending of AI with peer/group-based sociocultural learning.
- More clearly articulate how our findings extend constructivist, sociocultural, and adaptive learning theories, offering practical and theoretical contributions specific to low-resource settings.

**E. Conclusion Analysis**

**Reviewer Suggestion**

Link conclusions more explicitly to research questions and findings.

**Author Response:**
We revised the conclusion to:

- Structurally map the answers to each of the three research questions, reinforcing the coherence of the study’s logic.
- Reiterate key findings with quantitative and qualitative support, highlighting how each contributes to the practical implementation of AI in ESL.
- Provide more nuanced implications, including:
  - The urgency for culturally responsive AI tools;
  - The need for scalable teacher training;
  - The ethical responsibilities involved in student data usage.

**Dear Reviewer #4**
We sincerely thank you for your thoughtful and constructive feedback on our manuscript. Your comments have helped us refine our work for greater clarity, methodological rigor, and coherence. The revised text for reviewer 4 is highlighted in BLUE. Below, we provide detailed responses to each of your observations.

**Strengths**

We appreciate your recognition of the study’s relevance, structural coherence, well-integrated literature review, and meaningful findings related to AI integration in a developing country context.

**Areas for Improvement**

**1. Clarity and Writing Style**

- **Comment:** Simplify lengthy sentences and correct grammatical issues.
- **Response:** We have thoroughly revised the manuscript to improve readability by shortening long sentences, correcting grammatical issues, and enhancing the flow of ideas. A professional editing tool and peer review were also used to ensure clarity and consistency.

**2. Abstract**

- **Comment:** Include specific quantitative data and structure the abstract around the three key issues.
- **Response:** The abstract has been revised to reflect the structure of the three research questions: (1) instructional practices, (2) student performance (with quantitative data: 71% gain for experimental, 23% gain for control), and (3) implementation challenges. Specific percentages, effect sizes, and statistical significance have been included to substantiate claims.

**3. Methodology**

- **Comment:** Clarify data collection procedures, instruments, participant details, and ethical considerations.
- **Response:** We have now elaborated on:
  - **Data Sources:** Classroom observations, FGDs, and pre-/post-tests (writing and vocabulary).
  - **Participant Details:** Five AI-trained ESL teachers and 100 students (randomly assigned into experimental and control groups), detailed in the Sampling Techniques section.
  - **Instrument Design:** Separate writing and vocabulary tests; structured observation protocol; FGD guides (Appendices A–C).
  - **Ethics:** We explicitly mention that ethical approval was obtained, informed consent was secured, and confidentiality was maintained.

**4. Data Analysis and Interpretation**

- **Comment:** Specify statistical methods and ensure all claims are evidence-based.
- **Response:** We have clarified the use of **paired-sample t-tests** and **effect sizes (Cohen’s d)**. Qualitative data were analyzed using **Braun & Clarke’s thematic analysis**, and triangulation was conducted using **FGDs**, **observations**, and **test results**. All claims have now been directly tied to empirical findings.

**5. Citations and References**

- **Comment:** Update references and ensure citation consistency.
- **Response:** We have added several recent studies from 2023–2025 (e.g., Benek, Fan & Zhang, Rana et al.) and reviewed all references for consistency and completeness. In-text citations were also cross-checked to ensure alignment with the reference list.

**Additional Comments**

**1. Sector-Specific AI Applications**

- **Response:** We have now included specific examples of AI tools used in **education (Grammarly, Duolingo, Quizlet)**. While our primary focus is ESL, we have briefly contextualized AI relevance in other sectors (e.g., government and healthcare) in the Introduction.

**2. Comparative Discussion**

- **Response:** The Discussion section now includes a direct comparison with prior studies from both global and regional contexts, highlighting how our findings extend current knowledge, particularly through localized, empirical data from Pakistan.

**3. Real-World Examples**

- **Response:** The revised manuscript includes classroom-based cases and examples from FGD transcripts (e.g., challenges using AI in rural schools, Grammarly flagging culturally appropriate expressions) to illustrate practical implications.

**Final Note**

We thank you again for your invaluable insights, which have helped us strengthen the manuscript’s scholarly quality. We believe the revisions have significantly enhanced its clarity, depth, and contribution to the field of AI-integrated language education in low-resource settings.

Sincerely,
**Dr. Tahir Saleem (Corresponding Author)**
on behalf of all co-authors
