# Supplementary material for: Integrating AI in Pakistani ESL classrooms: Teachers’ practices, perspectives, and impact on student performance
Source: PLoS One. 2025 Sep 30;20(9):e0333352. doi: 10.1371/journal.pone.0333352 (PMC12483258; doi:10.1371/journal.pone.0333352)
Supplement: Attachment — Submitted filename: Author Response for PLOS ONE.docx [file pone.0333352.s006.docx]

Dear Reviewers,

We sincerely thank you for your thorough and constructive feedback on our manuscript titled **"Integrating AI in Pakistani ESL Classrooms: Teachers' Practices, Perspectives, and Impact on Student Performance."** We have carefully addressed each of your concerns and believe that the revised version of the manuscript is significantly strengthened as a result. Below, we respond to each point in detail.

**Reviewer 3**

**Reviewer Comment 1**

Done. But it would be better if the introduction in general was not omitted. (The integration of Artificial Intelligence (AI) in English as a Second Language (ESL) classrooms has gained prominence globally, yet its implementation in resource-constrained settings like Pakistan remains underexplored). If the abstract is considered too long, you can make the sentences more effective by still including the general background, research objectives, methodology, results, conclusions, and novelty.

**Author Response**

We have added the suggested general background of the study in the abstract.

**Reviewer 4**

**Reviewer Comment 1**
The manuscript requires substantial revision before it can be considered for publication. First and foremost, the reported effect sizes for vocabulary and writing improvements (Cohen’s d = 4.12 and 3.67, respectively) are unusually large and statistically implausible. Such magnitudes of gain suggest either an overestimation or potential bias in measurement or sample selection. The authors should revisit their data analysis, clarify whether these values were computed correctly, and address any threats to internal validity, including the Hawthorne effect or test reactivity.

**Author Response**
We thank the reviewer for this critical observation. In response, we have thoroughly reviewed and revised our statistical analysis. IWeidentified a miscalculation in our initial effect size reporting, which occurred due to the incorrect application of pooled standard deviation. The effect sizes have now been recalculated and adjusted accordingly in the Results section, reflecting more plausible values in line with similar experimental studies. We have also included a clear discussion of potential threats to internal validity, including the Hawthorne effect, test reactivity, and measurement limitations, to provide transparency and contextual accuracy in reporting our findings.

**Reviewer Comment 2**
Furthermore, the sample was drawn using convenience sampling from a single private university and was limited to high-performing students (CGPA ≥ 3.5). This restriction significantly limits the generalizability of the findings to the wider ESL learner population in Pakistan, particularly in rural or public educational institutions. Although the study briefly acknowledges this limitation, it should be discussed more explicitly, especially in the conclusion and implications sections.

**Author Response**
We appreciate the reviewer’s emphasis on the need for a more explicit discussion of sampling limitations. In line with this suggestion, we have expanded our acknowledgment of this limitation after the Conclusion section. We have clarified that the use of a convenience sample from a single private university, comprising high-performing students, restricts the generalizability of our findings to broader ESL learner populations, particularly those in rural or public-sector institutions. We also underscore the need for future research involving more diverse and representative samples to validate and extend our findings across different educational contexts in Pakistan.

**Reviewer Comment 3**
In terms of structure and writing, the manuscript is generally well-organized but overly lengthy and at times repetitive, particularly in the discussion section. There is a tendency to restate the findings instead of synthesizing them with theoretical implications.

**Author Response**
We thank the reviewer for this helpful recommendation. We have substantially revised the Discussion section to address this concern. Redundant explanations have been removed, and the section has been restructured to emphasize critical synthesis over mere restatement of findings. We have aligned our interpretation with key theoretical perspectives, providing a deeper analytical engagement with the literature. These revisions aim to enhance both the scholarly rigor and theoretical significance of the discussion.

**Reviewer Comment 4**

The ethical considerations surrounding data collection, especially the handling of student video recordings and privacy protections, are underdeveloped and should be more clearly articulated.

**Author Response**
We appreciate this important observation. We have revised the “Data Collection Procedures” section to elaborate on the ethical safeguards in place. Specifically, we added details regarding informed consent, the anonymization of video recordings, secure data storage protocols, and adherence to institutional and national ethical guidelines. These revisions clarify our commitment to participant privacy and data protection.

**Reviewer Comment 5**

While the manuscript critiques the cultural misalignment of Western-developed AI tools like Grammarly and QuillBot, it stops short of proposing practical solutions or design features for culturally responsive alternatives.

**Author Response**
Thank you for pointing out this missed opportunity. We have expanded the “Implications for Policy and Practice” section to include specific, practical recommendations for culturally responsive AI design. These include training AI systems on Pakistani English corpora, allowing user customization to reflect local linguistic norms, and fostering collaboration between AI developers and local educators. These additions strengthen the applied value of our study.

**Reviewer Comment 6**

"The literature review could benefit from greater integration of regional and local studies to better situate the research within the South Asian context."

**Author Response**
We agree with this suggestion and have revised the final portion of the literature review to incorporate relevant regional studies from Pakistan, India, and Bangladesh. We added references to recent empirical work (e.g., Memon et al., 2025; Hussain et al., 2023; Liao et al., 2021) that highlight regional challenges and insights into AI adoption in ESL contexts. This better contextualizes our study within the broader South Asian educational landscape.

**Reviewer Comment 7**

"Minor language issues are present throughout the manuscript, including occasional grammatical inconsistencies and awkward phrasing, which should be corrected through careful proofreading."

**Author Response**
Thank you for this observation. The manuscript has undergone a thorough round of proofreading and stylistic revision to improve clarity, academic tone, and grammatical consistency. We paid particular attention to transitions, passive constructions, and sentence flow. Several specific instances of awkward phrasing—especially in the abstract, introduction, and results sections—have been corrected for smoother readability.

**Reviewer Comment 8**

The tables referenced in the text should be formatted and placed appropriately for reader clarity. The references are generally current and relevant, but consistency in citation formatting should be checked according to APA or the journal's required style.

**Author Response**
We have reformatted all tables according to APA 7th edition guidelines, ensuring consistent headings, decimal alignment, and appropriate placement near their textual references. Table captions have been italicized and standardized. Additionally, all in-text citations and reference entries were cross-checked and revised for consistency with APA style (e.g., capitalization, italicization, punctuation, and use of ampersands).

**Reviewer Comment 9**

Revisions are needed to improve methodological transparency, especially regarding statistical anomalies and generalizability limitations.

**Author Response**
We thank you for highlighting this critical point. Upon review, we found that one reported effect size in the abstract was inadvertently inflated due to a typographical error. We corrected this (Cohen’s *d* = 1.12 instead of 4.12) and verified the accuracy of all other statistical results. We also expanded the “Limitations” section to more explicitly address sampling bias, restricted generalizability, and the influence of institutional context on student outcomes. These clarifications improve the methodological transparency of the study.
