# Supplementary material for: Integrating AI in Pakistani ESL classrooms: Teachers’ practices, perspectives, and impact on student performance
Source: PLoS One. 2025 Sep 30;20(9):e0333352. doi: 10.1371/journal.pone.0333352 (PMC12483258; doi:10.1371/journal.pone.0333352)
Supplement: Attachment — Submitted filename: Latest Rebuttal for PLOS ONE.docx [file pone.0333352.s007.docx]

Dear Editor,

I am writing regarding the recent review report received for my manuscript entitled *“Integrating AI in Pakistani ESL Classrooms: Teachers’ Practices, Perspectives, and Impact on Student Performance”* .

All reviewers, with the exception of Reviewer 4, have recommended the publication of our manuscript. Upon thorough examination of Reviewer 4's feedback, it appears that the report pertains to a different study and does not accurately reflect the scope, methodology, or content of our submission. I respectfully present the following points for your consideration:

1. **Terminology mismatch:** The reviewer refers to “thematic synthesis,” “three-dimensional engagement,” and “seven influencing factors.” My study employs thematic *analysis* but not thematic synthesis, and no such engagement framework or “seven influencing factors” is used in my work.
2. **Methodological mismatch:** The report critiques the absence of inter-rater reliability (e.g., Cohen’s Kappa) for article screening, the exclusion of non-English and grey literature, and the lack of publication bias mitigation. These concerns are relevant to a systematic review or meta-analysis using PRISMA guidelines, whereas my study is an **empirical mixed-methods classroom intervention** involving AI-assisted ESL instruction in Pakistan.
3. **Framework mismatch:** The reviewer references “Ellis’s” and “Han & Hyland’s” frameworks, as well as a suggested matrix mapping feedback types (teacher, peer, automated) to engagement dimensions. My research does not investigate feedback-type comparisons or adopt these theoretical models; instead, it evaluates the pedagogical integration of AI tools such as Grammarly, QuillBot, Quizlet, and Memrise in actual classroom practice.
4. **Scope mismatch:** The comments recommend exploring cross-cultural variation in “feedback acceptance” and integrating engagement-type mappings, which are unrelated to my study’s objectives, data, and design. My work focuses specifically on measurable vocabulary and writing gains and on teachers’ and students’ perceptions within Pakistani ESL classrooms.

Given these discrepancies, the review report appears to be intended for another manuscript. Furthermore, the language, structure, and generic breadth of the comments strongly suggest that the report may have been generated with the assistance of AI tools. If that is the case, I respectfully request that the editorial office sensitise reviewers to avoid submitting uncritical AI-generated reports, as such feedback risks misrepresenting the work under review and undermines the peer-review process. For completeness, I would like to highlight that all the comments from Reviewer 4 were meticulously incorporated into the revised manuscript, ensuring that the feedback was addressed comprehensively and constructively.

Thank you very much for your attention to this matter and for your continued efforts in maintaining rigorous scholarly standards.

Sincerely,
Tahir Saleem
Professor, Department of English
University of Central Punjab, Lahore, Pakistan
Email: tahir.saleem@ucp.edu.pk
